# Dependence On Glycolysis Sensitizes BRAF-mutated Melanomas For Increased Response To Targeted BRAF Inhibition

**DOI:** 10.1038/srep42604

**Published:** 2017-02-16

**Authors:** Keisha N. Hardeman, Chengwei Peng, Bishal B. Paudel, Christian T. Meyer, Thong Luong, Darren R. Tyson, Jamey D. Young, Vito Quaranta, Joshua P. Fessel

**Affiliations:** 1 Department of Cancer Biology, Vanderbilt University School of Medicine, 2220 Pierce Avenue, Nashville, TN 37232, USA; 2 Center for Cancer Systems Biology at Vanderbilt, Vanderbilt University School of Medicine, 2220 Pierce Avenue, Nashville, TN 37232, USA; 3 Chemical and Physical Biology Graduate Program, Vanderbilt University, Nashville, TN, 37232, USA; 4 Departments of Medicine & Pharmacology, Division of Allergy, Pulmonary, and Critical Care Medicine, Vanderbilt University School of Medicine, 21st Avenue South, Nashville, TN 37232, USA; 5 Departments of Chemical Biomolecular Engineering, and Molecular Physiology & Biophysics, Vanderbilt University, Nashville, TN 37232, USA.

## Abstract

Dysregulated metabolism can broadly affect therapy resistance by influencing compensatory signaling and expanding proliferation. Given many BRAF-mutated melanoma patients experience disease progression with targeted BRAF inhibitors, we hypothesized therapeutic response is related to tumor metabolic phenotype, and that altering tumor metabolism could change therapeutic outcome. We demonstrated the proliferative kinetics of BRAF-mutated melanoma cells treated with the BRAF inhibitor PLX4720 fall along a spectrum of sensitivity, providing a model system to study the interplay of metabolism and drug sensitivity. We discovered an inverse relationship between glucose availability and sensitivity to BRAF inhibition through characterization of metabolic phenotypes using nearly a dozen metabolic parameters in Principle Component Analysis. Subsequently, we generated rho0 variants that lacked functional mitochondrial respiration and increased glycolytic metabolism. The rho0 cell lines exhibited increased sensitivity to PLX4720 compared to the respiration-competent parental lines. Finally, we utilized the FDA-approved antiretroviral drug zalcitabine to suppress mitochondrial respiration and to force glycolysis in our cell line panel, resulting in increased PLX4720 sensitivity via shifts in EC50 and Hill slope metrics. Our data suggest that forcing tumor glycolysis in melanoma using zalcitabine or other similar approaches may be an adjunct to increase the efficacy of targeted BRAF therapy.

Melanoma is the most malignant form of skin cancer, and roughly 50% of clinical isolates have a mutation in the BRAF kinase of the mitogen-activated protein kinase (MAPK) pathway^1^,^2^. Ninety percent of those BRAF mutations are missense mutations that change the valine at position 600 to glutamic acid (V600E) or aspartic acid (V600D)^3^. The mutation confers constitutive activation of the BRAF kinase and drives oncogenic signaling through MEK phosphorylation. Targeted therapies against the mutant BRAF have prolonged progression-free survival and overall survival in Phase III clinical trials^4^. Unfortunately, most patients will exhibit some degree of disease progression while treated with a BRAF inhibitor, with nearly 50% of patients progressing after only 6 to 7 months of initial treatment^5^. There have been a variety of mechanisms that underlie initial and acquired drug resistance described in the literature. Generally, mechanisms of resistance to anti-BRAF therapies are put into MEK-dependent and MEK-independent categories. MEK-dependent mechanisms include mutations in NRAS, MEK1 and MEK2^6^, loss of RAS regulation by NF1^7^,^8^, COT overexpression driving MEK signaling^9^, and genetic alterations in BRAF itself, such as truncation or amplification^10^. MEK-independent mechanisms of resistance include receptor tyrosine kinase protein and ligand overexpression, such as cMET, IGF1R, and PDGFRβ^6^, and signaling through PI3K^11^. Unfortunately, more than 40% of the resistance found in patients who progressed on targeted therapy cannot be attributed to any of these mechanisms^12^. One of the features common to all of the known pathways that contribute to resistance is that they exert direct or indirect control of multiple cellular metabolic pathways—contributing to the single “hallmark” of metabolic reprogramming. In the last several years, there has been an increasingly intense focus on tumor metabolism as an exploitable therapeutic avenue^13^,^14^,^15^,^16^, with the success of asparaginase in the treatment of acute lymphoblastic leukemia (ALL) being just one example that has achieved widespread clinical use^17^,^18^, and with many other metabolism-based therapies under active development^19^,^20^.

Dysregulated metabolism in cancer has been shown to affect treatment outcome via multiple pathways, including the activation of compensatory receptor tyrosine kinase signaling to bypass molecular targeted therapies, the repression of pro-apoptotic signaling, and limitation of drugs’ access to molecular targets through active and passive mechanisms^20^. Komurov *et al*. showed chronic lapatinib treatment of HER2+ breast cancer cell lines produced cells with an advanced nutrient starvation phenotype^21^. Furthermore, the cells were sensitive to the antihelminthic pyrvinium pamoate, which targets mitochondrial function under various conditions^22^, particularly glucose deprivation^23^. Recently, it has been shown in BRAF-mutated melanoma that chronic treatment with BRAF inhibitor induces glutamine dependence that correlates with drug resistance^24^,^25^. We were interested in the prospect that the molecular metabolic landscape of any individual tumor might have a direct relationship to its sensitivity to targeted therapies.

The same metabolic pathways that have been targets for investigation in other malignancies have also been explored in BRAF-mutated melanoma, but a consensus of the major metabolic program exhibited by BRAF-mutated melanomas, or even whether a single dominant metabolic program exists, is lacking. BRAF-mutated melanomas have conversely been characterized as exhibiting primarily aerobic glycolysis^26^ or oxidative phosphorylation^27^,^28^. Moreover, the relationship between metabolic program and therapeutic response in BRAF-mutated melanoma is poorly understood, so we set out to probe the phenotypic relationship of metabolism and responses to the BRAF inhibitor vemurafenib.

In the present study, we used a panel of human BRAF-mutated melanoma cell lines to demonstrate *in vitro* variability in response to PLX4720, a BRAF inhibitor and analogue of vemurafenib. Utilizing our previously described method for measuring proliferative rate under various treatment conditions^29^, we calculated a metric describing the dependence of proliferation on drug concentration to place the cell lines on a continuum of sensitivity to PLX4720. We then examined baseline glycolytic and oxidative metabolism and found a relationship between reliance on glycolysis and sensitivity to inhibition by PLX4720. Building upon this observation, we show that forcing exclusive reliance on glycolysis via mitochondrial DNA depletion using either ethidium bromide or zalcitabine (a first generation antiretroviral used to treat HIV) significantly attenuates intrinsic resistance to PLX4720 in our cell line panel.

## Results

### BRAF-mutated Melanoma Cell Lines Exhibit Heterogeneous Oncogene-Inhibition Responses And Metabolic Strategies

**Figure 1:**
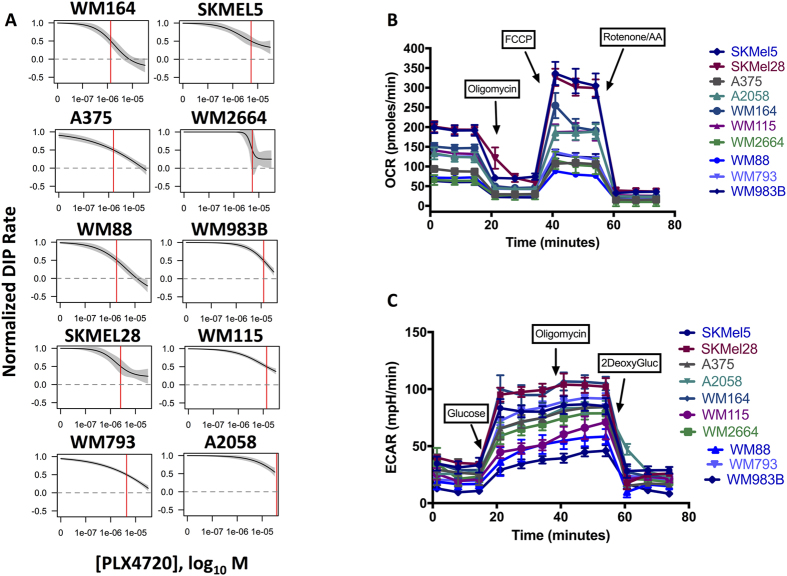
BRAF-mutated melanomas exhibit heterogeneous metabolic programs and responses to BRAF inhibition. (**A**) Proliferative spectrum of IC50’s for PLX4720 based on DIP rate. The dose-response curves are generated using a 2-fold dilution of PLX4720 from 32 uM down to zero (DMSO). The proliferative rates are calculated using the slope of the log2-normalized population curve after 48 hours (see Methods). (**B**) Oxygen consumption profiles for the cell lines with sequential additions of oligomycin (1 uM), FCCP (1 uM) and Rotenone/Antimycin A (0.5 uM). (**C**) Extracellular pH profiles for the cell lines with sequential additions of glucose (10 mM), oligomycin (1 uM), and 2-deoxyglucose (0.5 uM).

To confirm the variability in response to mutant BRAF inhibition observed in patients could be modeled *in vitro*, we measured the cell lines’ proliferative responses to BRAF inhibition. The proliferative kinetics of the cell lines were quantified in the presence of PLX4720. Based on the PLX4720-treated DIP rates, BRAF-mutated melanoma cell lines fall along a response spectrum or continuum (Fig. 1A), from highly sensitive (e.g., WM164) to largely insensitive (A2058). The IC50 metric is calculated from a log-logistic curve fit to the estimated rate of proliferation obtained at each drug concentration, known as the drug-induced proliferation (DIP) rate^29^.

We next wanted to confirm that the measured variability in response to PLX4720 treatment was not due to phenotypic selection of drug-resistant subclones during the short 4–5 day timeframe of our experiments. We leveraged a fluorescent ubiquitin-dependent cell cycle indicator (FUCCI; mAG-gem1-110) to detect cells that have committed to cell division (i.e. passed the G1/S transition). We reasoned that if intrinsically resistant clones exist within the population, they would be enriched in cells that continue to proliferate in the presence of BRAF inhibition and would remain resistant after isolation. To test this, we treated the BRAF-mutated melanoma lines with PLX4720 or DMSO for 72 hours followed by flow sorting for the actively dividing FUCCI+ cells from both groups, then re-plated them in the absence of drug for 24 h and treated a second time with PLX4720. The proliferative responses of the two groups were essentially the same, indicating that PLX4720 does not appear to select for resistant populations in the short term (Supplemental Fig. S1). Stated differently, cells that actively divide in the presence of PLX4720 have similar proliferation kinetics when re-challenged with the drug.

To determine the metabolic profiles exhibited by our panel of BRAF-mutated melanomas, we quantified lactate-producing glycolysis and mitochondrial oxidative metabolism using the Seahorse extracellular flux analyzer platform. Using a panel of 10 BRAF-mutated melanoma cell lines, we found most lines can variably utilize glucose and consume oxygen as part of mitochondrial respiration (Fig. 1B and C). Notably, most cells have minor glycolytic reserve after the addition of oligomycin (Fig. 1C), indicating most of the melanoma cell lines are functioning at or near their glycolytic capacity. The basal respiration and oxygen consumption also varied across cell lines. Additionally, the subsequent decreases in oxygen consumption rate (OCR) after the addition of oligomycin (Fig. 1B) suggest varying dependencies on ATP-linked respiration (or ATP turnover supported by oxidative phosphorylation) across the cell lines. In totality, these data suggest broad, intrinsic metabolic heterogeneity across the cell line panel.

### Metabolic Phenotype Variability Correlates With Variability In PLX4720 Response

**Figure 2:**
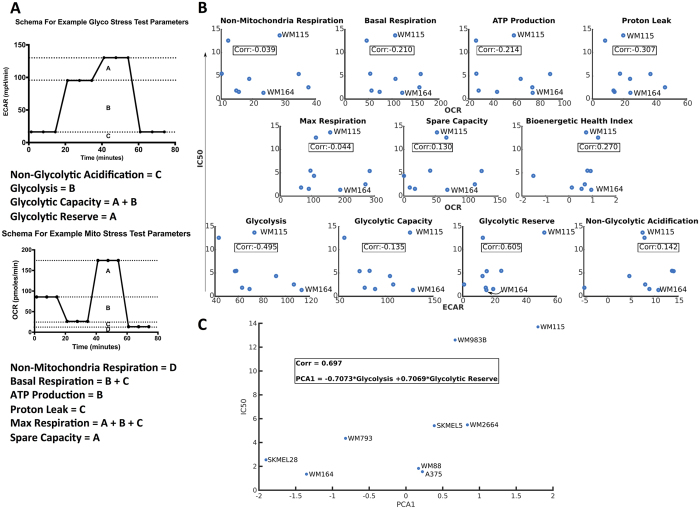
(**A**) Schematic diagram describing the components and metrics from Seahorse assays used in the PCA; Bioenergetic Health Index (BHI) calculation is described in methods. (**B**) Metabolic parameters individually tested for correlation (Pearson) to PLX4720 IC50 of the cells. (**C**) The first principle component of a linear combination of glycolysis and glycolytic reserve correlates with IC50.

Given the observed heterogeneity in PLX4720 responses without an obvious biological mutational trend (Fig. 1A and Supplemental Table 1) and the variable metabolic strategies employed by our panel of BRAF-mutated melanomas, we sought to examine more closely whether a direct relationship exists between metabolism and drug response. To quantify the relationship between the metabolic program of BRAF-mutated melanoma cell lines and PLX4720 response, eleven metabolic parameters were calculated from measurements of mitochondrial oxygen consumption and glycolytic function curves for nine cell lines (parameters described in Fig. 2A schema). Each metabolic parameter was independently tested for correlation with the measured IC50 for PLX4720 for each cell line (Fig. 2B). We found a significant inverse correlation (r = −0.495) between glycolysis and the measured IC50 values, suggesting increased glycolysis in BRAF-mutant cell lines is indicative of greater sensitivity to BRAF inhibition (and thus a lower IC50).

Next we determined how different combinations of the metabolic parameters correlated with drug sensitivity using Principle Component Analysis (PCA). This analysis comparing all possible combinations of parameters identified a linear combination of glycolysis and glycolytic reserve as strongly correlating to the cell line’s IC50 values, with the combination of these two metabolic parameters accounting for more than two-thirds (69.7%) of the variance in the parameter ensemble across the cell line panel (Fig. 2C). Based on these results, we predicted increasing the rate of glycolysis while depleting glycolytic reserve would decrease the IC50 value for a BRAF-mutated melanoma to PLX4720 treatment.

### Glucose is a Key Nutrient Influencing Response to Targeted BRAF Inhibition

We titrated across serial dilutions of PLX4720 and glucose concentrations to test whether glucose availability and PLX4720 response were functionally related. We first quantified drug-induced proliferative (DIP) rates across the spectrum of glucose/PLX4720 conditions in 6 melanoma cell lines. We found that the DIP rate responses are largely linear and glucose/PLX4720 concentration dependent, as shown in a heatmap (Supplemental Fig. S2). Glucose-replete conditions exhibited the “best” or highest DIP rates for each cell line (indicating more rapid cell proliferation), and the glucose-deprived conditions the poorest or lowest DIP rates, as might have been expected. The key finding was each cell line appears to have the capacity to revert phenotypically to a more PLX4720-sensitive phenotype (i.e. lower DIP rate) by simply lowering the glucose in the medium, and every cell line exhibited sensitivity to glucose limitation to approximately the same degree.

Using proliferation as a phenotypic output, every cells’ phenotype is pushed into a more responsive zone of lowered proliferation. These data suggest that glucose is a key nutrient tied to BRAF inhibition, and PLX4720 efficacy is maximized in glucose-limiting conditions.

### An Exclusively Glycolytic Metabolic Program Increases Sensitivity to BRAF Inhibition

**Figure 3:**
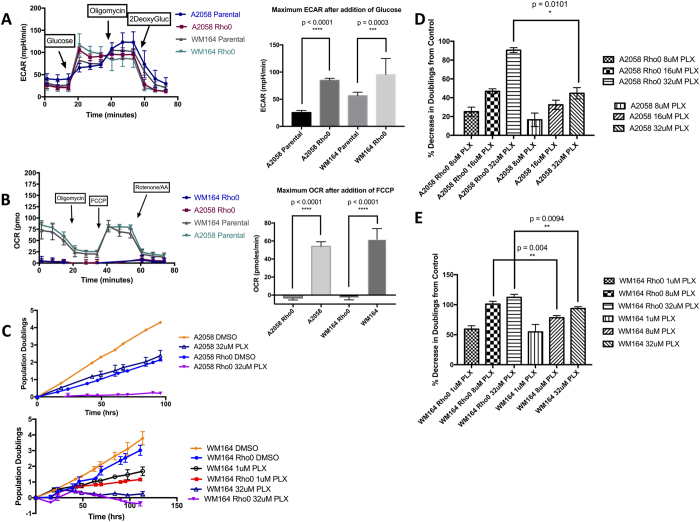
(**A**) Extracellular pH or acidification rate of parental A2058 and WM164, and their Rho0 derived counterparts; bar plot showing maximum ECAR after adding glucose (10 mM), with t-test between parental and Rho0. (**B**) Oxygen consumption rate of parental A2058 and WM164, and their Rho0 derived counterparts; bar plot showing maximum OCR after adding FCCP (1 uM), with t-test between parental and Rho0. (**C**) Log2 normalized proliferation of parental A2058 treated with DMSO or 32 uM PLX4720, and Rho0 A2058 treated with DMSO or 32 uM PLX4720 (purple). Similar population doublings-time plot of WM164 (lower panel), with additional dose of 1 uM PLX4720. (**D**,**E**) Quantification of percent decrease in Doublings from respective DMSO control (ie., parental compared to parental, or Rho0 compared to Rho0).

To test the hypothesis that increased glycolytic dependence is functionally related to enhanced PLX4720 response, we generated rho0 variants of two of our melanoma lines, A2058 and WM164. These two lines were chosen because they represented the least and most sensitive lines, respectively, to PLX4720 in our assays. Generating rho0 variants (which lack mitochondrial DNA and, thus, lack a functional electron transport chain) allowed assurance of a quantitative shift to an exclusively glycolytic metabolic program. We confirmed depletion of mitochondrial DNA (data not shown) and showed absence of mitochondrial oxygen consumption (Fig. 3B) and a significant increase in glycolytic rate combined with a significantly decreased glycolytic reserve (Fig. 3A) compared to the parent line for each variant. Rho0 cells have been shown to be capable of apoptosis^30^,^31^ and are largely reported as carrying “ghost” mitochondria that lack electron transport chain functionality. Using the rho0 cells as a model, we tested whether forced glycolysis impacts response to BRAF inhibition by treating the rho0 variants with increasing doses of PLX4720. We first noted that, as might be expected, the overall proliferative rates of the rho0 cells were decreased compared to their parental counterparts (Fig. 3C). However, even taking this into account, the percent inhibition (based on the final population doublings) with PLX4720 treatment was significantly increased in the A2058 & WM164 rho0 cells compared to their parental cell lines (Fig. 3D and E).

Mitochondrial respiration provides more than just ATP through oxidative phosphorylation, as rho0 cells and electron deficient cells can still proliferate if provided uridine and pyruvate^32^,^33^,^34^. To test whether increased PLX4720 sensitivity in the A2058 rho0 line might be due to a deficiency in the necessary biosynthetic intermediate aspartate, which is normally produced through mitochondrial respiration^35^,^36^ we supplemented both pyruvate and exogenous aspartate to attempt to rescue the A2058 rho0 cells. Not only did this not rescue the proliferative response, proliferative rates were actually further decreased upon treatment with PLX4720 (Supplemental Fig. S3).

**Figure 4:**
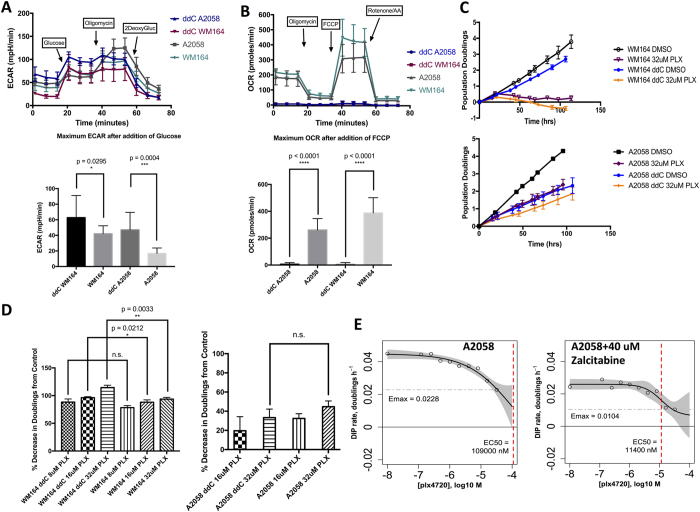
(**A**) Extracellular pH or acidification rate of parental A2058 and WM164, and their ddC/zalcitabine treated counterparts (40 uM zalcitabine); bar plot showing maximum ECAR after adding glucose (10 mM), with t-test between parental and ddC treated cells. (**B**) Oxygen consumption rate of parental A2058 and WM164, and their ddC/zalcitabine treated counterparts (40 uM zalcitabine); bar plot showing maximum OCR after adding FCCP (1 uM), with t-test between parental and ddC treated cells. (**C**) Log2 normalized proliferation of ddC treated WM164 (top panel) or ddC treated A2050 (lower panel). (**D**) Quantification of percent decrease in Doublings from respective DMSO controls for WM164 and A2058 cell line (not statistically significant). (**E**) Drug Induced Proliferative (DIP) metric dose response curves (non-normalized) marking model estimated EC50 values in nM (red dashed vertical line), and model estimated Emax values (grey dashed horizontal line).

Finally, we sought to replicate these findings and to force glycolysis using an alternative method with greater translational potential than ethidium bromide treatment. To accomplish this, we utilized the FDA-approved antiretroviral drug zalcitabine, also called ddC, in our cell lines. Zalcitabine/ddC is a nucleoside analog utilized as a first generation antiretroviral in the treatment of HIV^37^, falling out of favor largely due to the mitochondrial toxicities it exerted on various tissues and organs and the development of less toxic and more effective antiretrovirals^38^,^39^. In humans, ddC has been shown to deplete mitochondrial DNA^40^,^41^, and *in vitro* assays have been utilized with up to 300 uM ddC^38^, with efficient mtDNA depletion typically observed at concentrations in the 10–50 uM range. In our melanoma cell lines, treatment with 40 uM ddC phenocopied the suppression of mitochondrial oxygen consumption (Fig. 4B), the increase in glycolysis, and the reduction in glycolytic reserve (Fig. 4A) seen in the rho0 cell lines. Utilizing our previous model for *in vitro* assays^29^, we found the PLX4720 response in ddC-treated WM164 was significantly increased: the proliferative kinetics phenocopied the prior WM164 rho0 experiments and the percent decrease in doublings was statistically significant (Fig. 4C upper panel, Fig. 4D, left panel). In contrast, the PLX4720-resistant line A2058 was more substantially affected by the attenuation of proliferation associated with ddC treatment itself: the percent decrease in doublings was not significant (Fig. 4D, right panel). However, the effect on proliferative kinetics of a single ddC treatment phenocopied the high-dose PLX4720 treatment (Fig. 4C, lower panel). Moreover, ddC treatment in A2058 reduced the EC50 for PLX4720 ten-fold and also substantially reduced Emax (Fig. 4E), both effects consistent with significantly enhanced efficacy of PLX4720 in the context of ddC pretreatment. Additionally, the rate of proliferation affected the dose-response slopes, or Hill coefficients, revealing that ddC treatment decreased the Hill slope metric (Supplemental Fig. S4).

## Discussion

All cells, including tumor cells, have basic energy and metabolic needs for survival and proliferation. Cellular responses—such as adaptation, differentiation, proliferation, and signal transduction—are inherently complex and dynamic in nature. Therefore, sustained proliferation in the presence of targeted inhibitors is likely shaped by a cell’s dynamic metabolic constraints. In our study we investigated whether there exists a direct link between overall metabolic program and sensitivity to targeted BRAF inhibition, and if that relationship could be exploited to increase sensitivity in the cells. Our results demonstrate over-reliance on glycolysis can sensitize BRAF-mutated melanoma cells to targeted BRAF inhibitor treatment. The cells had significantly reduced proliferation (even death) when co-treated with PLX4720 and zalcitabine. This finding is in agreement with earlier reports^28^,^42^ suggesting mitochondrial inhibitors like oligomycin would be therapeutically beneficial in this cancer type. However, the use of oligomycin in humans would be difficult due to its extreme toxicity, and other mitochondrial inhibitors, like metformin and phenformin, probably exert antitumor effects but have potential dosage issues^43^ and lactic acidosis problems^44^, respectively. Antiretrovirals like zalcitabine, in contrast, have toxicities that may be easier to manage, and the portfolio of nucleoside analogs has been greatly expanded compared to the clinically available mitochondrial inhibitors^37^,^39^. Moreover, there is a tremendous amount of clinical experience with antiretrovirals used alone, in combinations, and in the context of many other drugs. The dose of ddC used in our studies is admittedly high in comparison to plasma concentrations typically achieved with conventional dosing to treat HIV infections, though our timeframe for treating cells to achieve mtDNA depletion was also very short. In translating these findings to *in vivo* studies, lower doses of ddC for longer periods of time would be expected to produce the same effects while allowing toxicities to remain manageable. The DIP rate metric overcomes time-related biases of slow acting vs. fast acting drugs, fast vs. slow-proliferating cells, and complex contributions of cell death and division that plague typical end-point assays. Interestingly, though, the Hill slope (or Hill coefficient) emerges as an important metric for evaluating antiretrovirals in the context of cancer therapeutics, and it has already been postulated by others to be clinically important when assessing drug sensitivity in the context of non-genetic influences^45^. Moreover, as a consequence of “washing out” time-dependent biases, the DIP rate metric may miss potentially clinically meaningful findings. This can be seen with ddC treatment of A2058, where a major effect of high dose, short duration ddC treatment is to slow proliferative rate. This is problematic for DIP rate-based assessments, but clinically, a slower growing tumor would generally be regarded more favorably than a rapidly growing one. Moreover, DIP rate analysis alone would miss the 10-fold shift in the EC50 for PLX4720 in ddC-treated A2058. Should this effect translate fairly directly into *in vivo* studies, this would represent a shift of EC50 for BRAF inhibitor from outside the typically achievable range into a dose range that is readily achievable and that may allow for lower doses to limit toxicities.

However, there still remain questions about what physiological role glycolysis, oxidative phosphorylation, or the mitochondria play for BRAF-mutated melanomas, particularly under the context of drug treatment. It can be speculated that BRAF inhibition cuts off the oncogenic signaling that is ramping up the metabolism and ATP production. ATP hydrolysis and glucose flux have been postulated to be intimately linked in highly proliferative cells: the ATP/AMP ratio is repressed and kept to a minimum by linking high ATP consumption activities (like N-glycosylation and folding of proteins^46^) with increased glycolytic flux for biosynthetic production. Thus, the high amounts of ATP generated from glycolysis are shuttled in order to relieve negative feedback inhibition on major glycolysis enzymes such as PFK. Other mechanisms to control ATP/AMP levels involve the tumor suppressor LKB1 and its target the AMP-activated Kinase: loss of function mutations and deletions of LKB1 in non-small cell lung cancers^47^ and Peutz-Jeghers Syndrome^48^, and activating mutations in PI3K and AKT that lead to strong signaling increasing glycolytic flux and ATP (and thus preventing the activation of AMPK through high levels of AMP)^49^,^50^,^51^. In BRAF-mutated melanomas it has been shown that the strong signaling down the MEK-ERK-RSK pathway enables negative regulation on LKB1 through phosphorylation of S325 and S428 sites^52^. Interestingly, WT BRAF immuno-precipitates with AMPK^53^ and high phospho-ERK staining inversely correlated with low phospho-AMPK staining *in vivo* under the context of WT BRAF^53^. To put these finding into context WT BRAF uses AMPK in the context of integrating energy metabolism with proliferation; mutant BRAFV600E will dampen the influence of AMPK, possibly because it is not needed for downstream metabolic signaling. BRAF inhibition may relieve the negative feedback regulation on LKB1 and thus, provide an avenue of metabolic rescue that would include mitochondrial biogenesis to make up for the resultant diminished ATP production. Therefore, in our work when we targeted mitochondrial DNA and rendered the cells functionally deficient, we metabolically constrained the cells to rely on glycolysis for all ATP (and redox regeneration).

Our approach of targeting differential tumor metabolism represents a break from previous models constrained within the more narrow scope of looking upstream and downstream along the RAS-RAF-MEK-ERK signaling axis. Identifying avenues of resistance through “non-oncogenic vulnerabilities” has been suggested^54^,^55^, particularly since many cancers are multifaceted and resistant to single target therapies. The functional integration of drug resistance mechanisms leads to adaptive, independently actionable phenotypes. Targeting the metabolic phenotype rather than a single genetic driver appears promising: the phenotype sustains the tumor–not necessarily the pathway, due to signaling plasticity and mechanistic redundancies. Thus, the benefit of targeting a terminal phenotypic state and bypassing the risk of oncogene switching or secondary mutations can be realized with current FDA-approved drugs such as zalcitabine. The strength of this study is that we used a large panel of cell lines, treated with physiologically relevant doses of the BRAF inhibitor PLX4720.

## Materials and Methods

### Reagents

PLX4720 was obtained from Selleck Chem. Glycolysis Stress Test and Mitochondrial Stress Test kits were obtained from Seahorse Biosciences and used according to manufacturer instructions. Ethidium bromide and uridine were obtained from Sigma. Dulbecco’s Modified Eagle Medium (catalog 11965-092) from Sigma.

### Cell Culture

Cells were grown and cultured in DMEM media containing 2 mM glutamine, 4.5 g/L glucose, 10% FBS and no sodium pyruvate (catalog 11965-092), except where specified otherwise. Cells were split and seeded at ratios that allowed for splitting 1–2× per week. For proliferative experiments, the cells were plated the night before, then reagents/drugs were prepared in fresh media and added to the cells immediately before the start of the experiment the following day. For experiments involving nutrient deprivation (like glucose deprivation), cells were washed 1× with PBS then experimental media was added onto the cells. For glucose deprivation, dialyzed FBS was added to DMEM medium to mitigate contribution of glucose from FBS. Dialyzed FBS+ No glucose DMEM was titrated against the normal FBS+ DMEM when preparing the drug dilutions for the experiments.

### Proliferation Assays

The cells were labeled lentivirally with a fluorescent, nuclear tag (Histone 2B monomeric Red Fluorescent Protein, H2BmRFP from AddGene), flow sorted for H2BmRFP positivity (top 10–15% brightest), and fluorescently counted under drug treatments. Cells were seeded into 96 well plates (1–5,000 cells per well) and drug treatments applied the following day, including DMSO or PBS control (all concentrations contained equal percentage of DMSO or PBS solvent). Images were taken every 8–12 hours with sufficient image alignment (montaging) in order to capture about 25–100 cells per well/treatment (over the course of the experiment, cell counts typically exceed 1,000 in DMSO or low drug concentration wells). Direct measurements of cell counts were made using Cellavista software and Image J macros. The images were filtered through these computer programs to track and label each cell, quantifying the number of cells in each time-stamped frame. Proliferation was plotted as log2 normalized growth, using the initial cell count from the first image frame for normalization.

### Rho0 cell lines

Rho0 cell variants of BRAF-mutated cell lines WM164 and A2058 were generated using DMEM medium containing 4.5 g/L or 25 mM glucose, 2 mM glutamine, 1 mM sodium pyruvate, 50 ug/ml uridine and 50 ng/ml ethidium bromide; cells were passaged at least 10× in this medium before using in experiments. PCR was used to confirm loss/reduction of mtDNA as a ratio of mtDNA to nuclearDNA (data not shown). These cells are classically referred to as rho0 or ρ0.

Measurement of Oxygen Consumption and Extracellular Acidification Rates Cells were plated in 96-well plates (Seahorse Biosciences, Bilerica, MA) at a density of 25–40,000 cells/well 24 hours before analysis on the Seahorse XF^e^ 96 extracellular flux analyzer. Mitochondrial oxygen consumption was quantified using the Mito Stress Test kit, and glycolytic rate quantified using the Glycolysis Stress Test kit, each according to manufacturer’s instructions. Briefly, assay medium was unbuffered DMEM containing either 10 mM Glucose, 2 mM Glutamine, and 1 mM Sodium Pyruvate (Mito Stress Test) or none of the aforementioned (Glyco Stress Test). No FBS was used in assay medium.

### Principal Component Analysis (PCA) and Liner Regressions

Metabolic parameters were extracted for nine cell lines from two representative experiments, a glycolytic function experiment (Glyco Stress Test) and a mitochondria function experiment (Mito Stress Test) according to equations in Supplemental Table. Bioenergetic Health Index was calculated as previously described by Chacko *et al*.^56^. Correlation between metabolic parameter and IC50 was calculated using Pearson correlation. Before principal component analysis (PCA), each extracted parameter was Z-score normalized to minimize variation due to the different parameter scales. The first principal component was calculated using all possible combinations of parameters and each combination was correlated with the measured IC50 for nine cell lines in panel. All code for analysis is available in the supplement.

### Statistical Analyses

Data are presented as either an average of 3+ separate experiments or a representative example; error bars are means+ or −SD and p values were obtained using unpaired t-test (Gaussian distribution assumed, two-tailed) done in Prism 7. Statistics for PCA and IC50 calculation are described in preceding sections.

## Additional Information

**How to cite this article**: Hardeman, K. N. *et al*. Dependence On Glycolysis Sensitizes BRAF-mutated Melanomas For Increased Response To Targeted BRAF Inhibition. *Sci. Rep.* **7**, 42604; doi: 10.1038/srep42604 (2017).

**Publisher's note:** Springer Nature remains neutral with regard to jurisdictional claims in published maps and institutional affiliations.

## Supplementary Material

Supplemental Figures
