# Supplementary material for: Dependence On Glycolysis Sensitizes BRAF-mutated Melanomas For Increased Response To Targeted BRAF Inhibition
Source: Sci Rep. 2017 Feb 16;7:42604. doi: 10.1038/srep42604 (PMC5311997; doi:10.1038/srep42604)
Supplement: Supplemental Figures [file srep42604-s1.pdf]

Supplemental Figures and Figure Legends

Title: Dependence On Glycolysis Sensitizes BRAF-mutated Melanomas For Increased Response To Targeted BRAF Inhibition

Authors: Keisha N. Hardeman[1,2], Chengwei Peng[1,2], Bishal B. Paudel[2,5], Christian T. Meyer[2], Thong Luong[4], Darren R. Tyson[1,2], Jamey D. Young[3], Vito Quaranta[1,2], and Joshua P. Fessel[1,4,*]

Author Affiliations:

[1]Department of Cancer Biology, Vanderbilt University School of Medicine, 2220 Pierce Avenue, Nashville, TN 37232

[2]Center for Cancer Systems Biology at Vanderbilt, Vanderbilt University School of Medicine, 2220 Pierce Avenue, Nashville, TN 37232

[3]Departments of Chemical Biomolecular Engineering, and Molecular Physiology & Biophysics, Vanderbilt University, Nashville, TN 37232

[4]Departments of Medicine & Pharmacology, Division of Allergy, Pulmonary, and Critical Care Medicine, Vanderbilt University School of Medicine, 21st Avenue South, Nashville, TN 37232

[5]Chemical and Physical Biology Graduate Program, Vanderbilt University, Nashville, TN, USA 37232

Supplemental Table 1

Table 1. BRAF-Mutated Melanoma Cell Lines Exhibit Heterogeneity

	Relevant Mutation/Biology			IC50 Metrics
**Cell Line**	**BRAF Mutation**	**P53 Status**	**PTEN Status**	**IC50 (uM)**
WM164	V600E	MU	WT	1.34
A375	V600E (HOMOZ)	WT	WT	1.55
WM88	V600E	WT	WT	1.83
SKMEL28	V600E (HOMOZ)	MU	MU	2.55
WM793	V600E	WT	MU/HEM DEL	4.35
SKMEL5	V600E	WT	WT	5.42
WM2664	V600D	WT	HEM DEL	5.49
WM983B	V600E	MU	WT	12.6
WM115	V600D	WT	HEM DEL	13.7
A2058	V600E	MU	MU	38.3

Table Legend:
Cell Line mutation information from WISTAR Institute website, COSMIC website, and and Birgit Schittek, et al., Int J. of Cancer; 82, 583-585 (1999)

Supplemental Figure S1

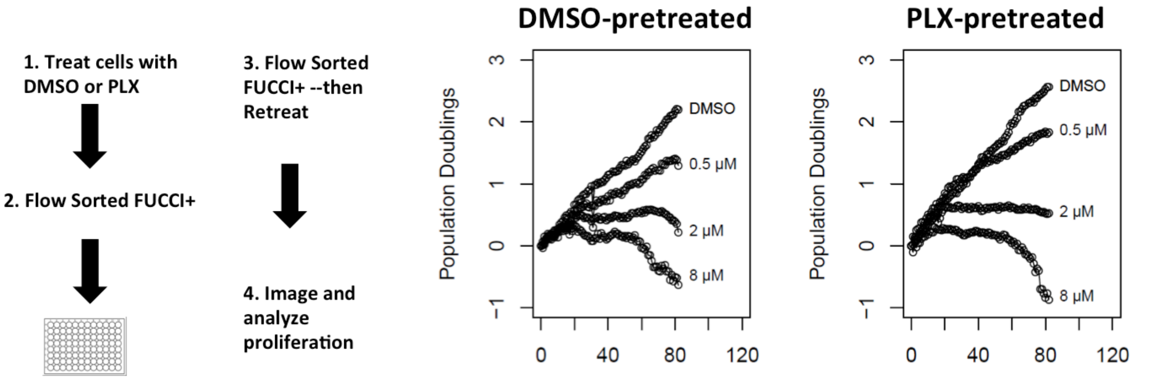

Figure Legend:
Schematic outlines the flow of the experimental design: the cells are pre-treated for 72 hours with either DMSO or PLX4720, then flow sorted for positive fluorescence-ubiquitin-linked cell cycle indicator (FUCCI). Those cells were seed in 96 well plates overnight, then retreated with PLX4720. The proliferation was log-2 normalized and plotted as population doubling (y-axis) over time (x-axis, in hours). The cell line used was A375.

Supplemental Figure S2

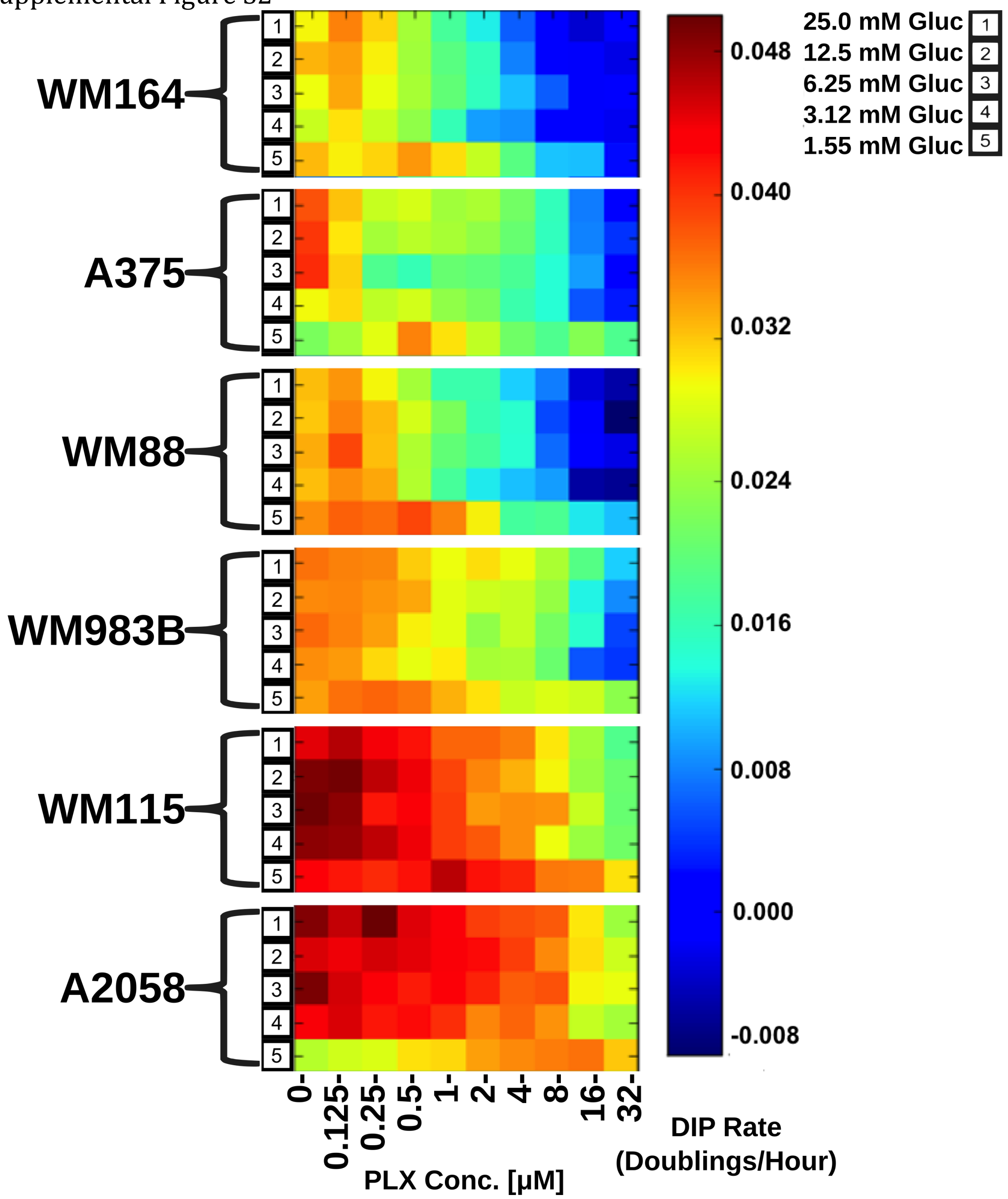

Figure Legend:

Un-scaled heatmap of the proliferative responses of cell lines treated in PLX4720 dose-response assay coupled with varying glucose concentrations (indicated in legend, 5 glucose/media conditions total). The doses of PLX4720 are the x-axis (10 doses, from 32 uM to zero/DMSO) and concentrations of glucose are the y-axis (from 25 mM to 1.55 mM, labeled using legend key).

Supplemental Figure S3

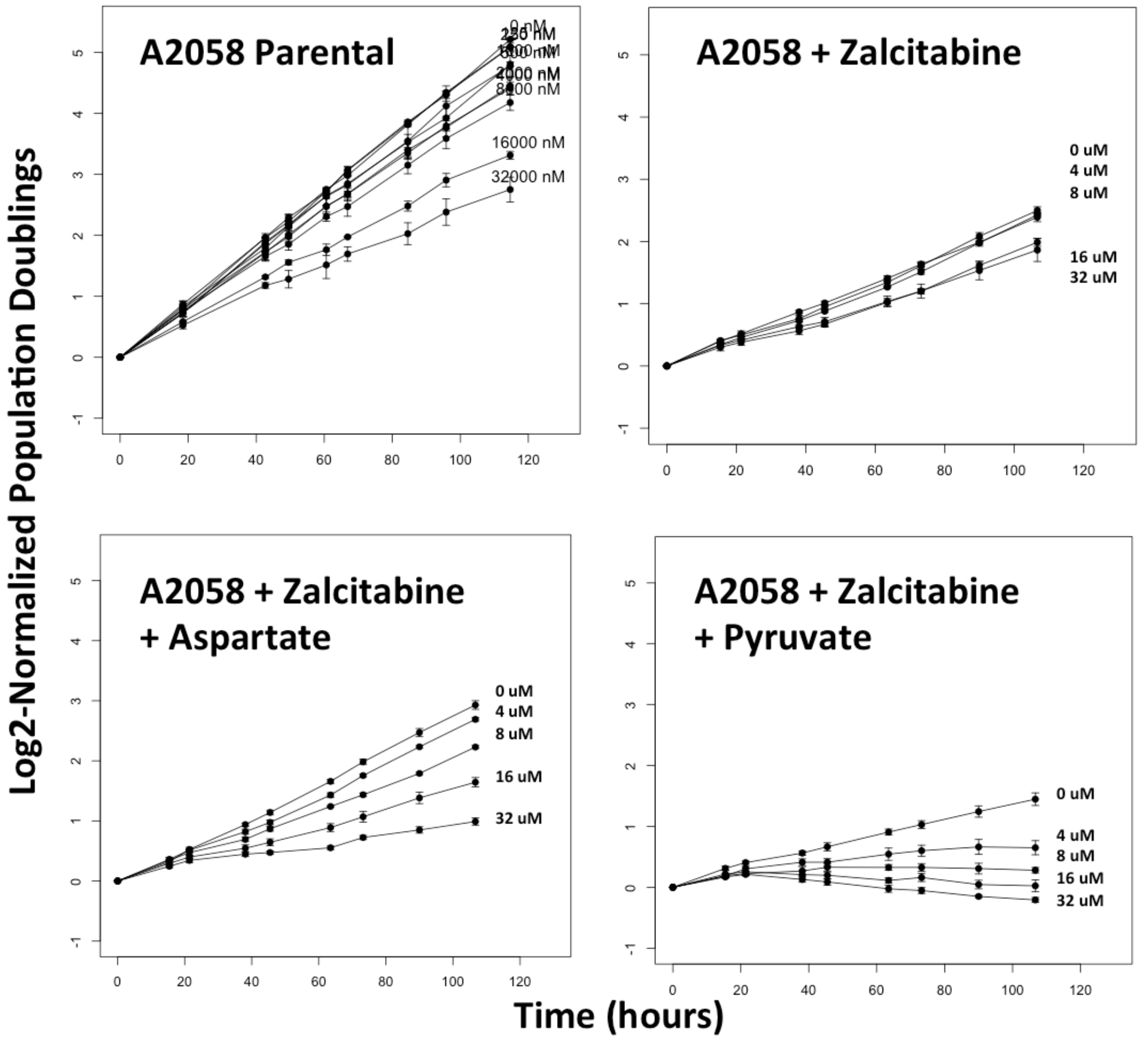

Figure Legend:
Log2-normalized proliferation of parental A2058 (top left plot) and pre-treated ddC/zalcitabine A2058 (remaining 3 plots, pre-treated with 40uM of zalcitabine for ~5 days). 10 mM Aspartate (bottom left) or 10 mM Sodium Pyruvate (bottom right) was added to the medium.

Supplemental Figure S4

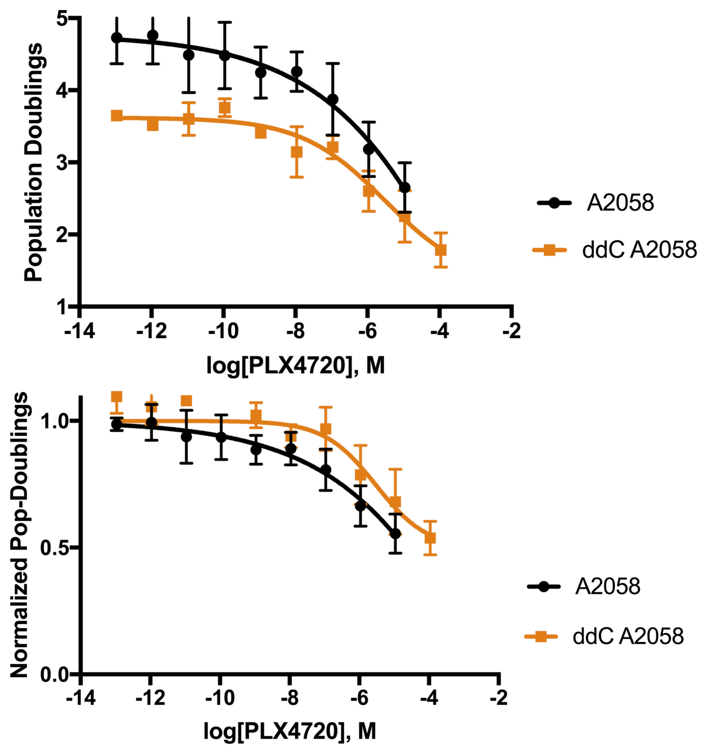

Figure Legend:
Non-normalized (upper panel) and normalized (lower panel) dose-response curves of A2058 parental and A2058 pre-treated with ddC.

Supplemental Matlab Code for PCA Analysis:

Code is available in the public repository in GitHub:

https://github.com/hardemkn/Hardeman_et_al_2016
